# 91 Precursors to Oliguria During Acute Burn Resuscitation: A Secondary Analysis of a Prospective Observational Study

**DOI:** 10.1093/jbcr/irad045.064

**Published:** 2023-05-15

**Authors:** Cody McHargue, Julie Rizzo, James Aden, Tam Pham

**Affiliations:** University of Washington, Seattle, Washington; Brooke Army Medical Center, La Vernia, Texas; Brooke Army Medical Center, San Antonio, Texas; University of Washington, Seattle, Washington; University of Washington, Seattle, Washington; Brooke Army Medical Center, La Vernia, Texas; Brooke Army Medical Center, San Antonio, Texas; University of Washington, Seattle, Washington; University of Washington, Seattle, Washington; Brooke Army Medical Center, La Vernia, Texas; Brooke Army Medical Center, San Antonio, Texas; University of Washington, Seattle, Washington; University of Washington, Seattle, Washington; Brooke Army Medical Center, La Vernia, Texas; Brooke Army Medical Center, San Antonio, Texas; University of Washington, Seattle, Washington

## Abstract

**Introduction:**

Though commonly used to tailor fluid resuscitation, urine output may be a late indicator of impending burn shock. We sought to identify precedents of an early oliguric episode within 6 hours of admission. We hypothesize that specific variables may indicate early at-risk features during resuscitation.

**Methods:**

We performed a secondary analysis of adults enrolled in the Burn Navigator resuscitation trial. We divided into two cohorts: those with oliguria (< 30ml/hr) and those without. Variables were compared by descriptive statistics.

**Results:**

A total of 146 adults met inclusion criteria. More oliguric patients experienced systolic pressures < 90 mmHg (p=0.02) or Diastolic pressures < 40 mmHg (P=0.01). The minimum bicarbonate level (p=0.04), larger TBSA (p=0.01) and larger full thickness component (p=0.004) were all predictors of early oliguria. There were also more female patients (p=0.003) in the oliguric group. No cohort difference was observed for these variables: burn mechanism, percent partial thickness, HR >140 beats per minute (BPM) or hourly change in HR >10 BPM, temperature < 36C, hourly MAP drop >10 mmHg, creatine kinase, age, body mass index, hematocrit level, alcohol level, or methamphetamine use.

**Conclusions:**

Specific variables and physiological derangement thresholds precedes the first oliguric episode during major burn resuscitation. These variables constitute at-risk features that may help derive future algorithms for efficient burn resuscitation.

**Applicability of Research to Practice:**

Precursor variables of an early oligouric episode identify patients needing closer fluid titration.

